# Transcription profile of soybean-root-knot nematode interaction reveals a key role of phythormones in the resistance reaction

**DOI:** 10.1186/1471-2164-14-322

**Published:** 2013-05-10

**Authors:** Magda Aparecida Beneventi, Orzenil Bonfim da Silva, Maria Eugênia Lisei de Sá, Alexandre Augusto Pereira Firmino, Regina Maria Santos de Amorim, Érika Valéria Saliba Albuquerque, Maria Cristina Mattar da Silva, Joseane Padilha da Silva, Magnólia de Araújo Campos, Marcus José Conceição Lopes, Roberto Coiti Togawa, Georgios Joanis Pappas, Maria Fatima Grossi–de–Sa

**Affiliations:** 1 Federal University of Rio Grande do Sul, Porto Alegre, RS 91501-970, Brazil; 2 Embrapa Genetic Resources and Biotechnology, Brasilia, DF 70770-917, Brazil; 3 Agricultural Research Company of Minas Gerais State, Uberaba, MG 38001-970, Brazil; 4 Federal University of Campina Grande, Cuité, PB 58175-000, Brazil; 5 University of Brasília, Brasília, DF 70910-900, Brazil; 6 Catholic University of Brasília, Brasília, DF 70790-160, Brazil

**Keywords:** Root–knot nematode, *Glycine max*, Transcriptome, Pyrosequencing, Plant–pathogen interaction, Hormone

## Abstract

**Background:**

Root-knot nematodes (RKN– *Meloidogyne* genus) present extensive challenges to soybean crop. The soybean line (PI 595099) is known to be resistant against specific strains and races of nematode species, thus its differential gene expression analysis can lead to a comprehensive gene expression profiling in the incompatible soybean-RKN interaction. Even though many disease resistance genes have been studied, little has been reported about phytohormone crosstalk on modulation of ROS signaling during soybean-RKN interaction.

**Results:**

Using 454 technology to explore the common aspects of resistance reaction during both parasitism and resistance phases it was verified that hormone, carbohydrate metabolism and stress related genes were consistently expressed at high levels in infected roots as compared to mock control. Most noteworthy genes include those encoding glycosyltransferases, peroxidases, auxin-responsive proteins and gibberellin-regulated genes. Our data analysis suggests the key role of glycosyltransferases, auxins and components of gibberellin signal transduction, biosynthesis and deactivation pathways in the resistance reaction and their participation in jasmonate signaling and redox homeostasis in mediating aspects of plant growth and responses to biotic stress.

**Conclusions:**

Based on this study we suggest a reasonable model regarding to the complex mechanisms of crosstalk between plant hormones, mainly gibberellins and auxins, which can be crucial to modulate the levels of ROS in the resistance reaction to nematode invasion. The model also includes recent findings concerning to the participation of DELLA-like proteins and ROS signaling controlling plant immune or stress responses. Furthermore, this study provides a dataset of potential candidate genes involved in both nematode parasitism and resistance, which can be tested further for their role in this biological process using functional genomics approaches.

## Background

Plant–parasitic nematodes rank among the most destructive group of plant pathogens and are extremely challenging to manage [1]. Several genera of nematodes parasitize soybean [*Glycine max (L.) Merrill*] worldwide, and the highest economic impact is attributed to root-knot nematodes (RKN), soybean cyst nematodes (SCN), lesion nematodes and reniform nematodes.

RKN are biotrophic parasites of the genus *Meloidogyne* and the most damaging species are *Meloidogyne incognita* and *M. javanica*, followed by *M. arenaria*. However, *M. javanica,* which is widespread in tropical regions, has become more aggressive than *M. incognita*[2]. Concerning RKN control, soybean faces the same economic losses and difficulties as other crops. Despite the use of management strategies such as crop rotation with non–hosts, sustainable and long–lasting pest control strategies are in high demand [3]. One of the strategies is to deploy novel sources of RKN resistance in soybean breeding programs, for example using the soybean line PI 595099 (Accession NPGS/GRIN: G93-9223), which is resistant against specific strains and races of nematode species, including *M. javanica, M. incognita, M. arenaria* and also the soybean cyst nematode *Heterodera glycines*[4]. Another alternative is to introduce genetic modifications in soybean plants to obtain RKN resistance. In both cases, it is important to elucidate the molecular mechanisms involved in RKN–soybean interactions.

Gene characterization has helped to clarify molecular mechanisms involved in plant defense that activate distinct responses [5,6]. New insights into the underlying defense signaling network regulated by hormones have been achieved through identification of key components and understanding the role of salicylic acid (SA), jasmonates (JA) and ethylene (ET) in plant responses to biotic stresses [7]. Recent studies indicate that other hormones such as abscisic acid (ABA), auxins (AUX), gibberellins (GAs), cytokinins (CKs), brassinosteroids (BR) and peptide hormones are also implicated in plant defense signaling pathways, but their role in plant defense is not very well known [8]. These phytohormones regulate the gene expression of plant defense and eventually trigger the production of defense molecules like phenylpropanoids [9], phytoalexins [10] and pathogenicity-like proteins (PR) [11].

GAs are well known for their function in controlling growth, although little is known about their effects on metabolic adjustments and influence on the fine-tuning release of reactive oxygen species (ROS) in response to biotic and abiotic stresses. Recently, comparisons made at the transcript and metabolite levels demonstrated that the variation in the GA regime affects growth by uncoupling it from carbon availability, which suggests that GA levels can affect plant primary metabolism [12]. These observations also revealed an interaction between energy metabolism and GA-mediated control of growth to coordinate cell wall extension, secondary metabolism, and lipid metabolism. Furthermore, it has been shown that GA can provide a mechanism for environmentally responsive growth regulation, causing ROS levels to remain low after biotic or abiotic stress [13]. This information suggests that changes in GA levels can couple the downstream regulation of growth and stress tolerance through modulation of ROS levels. Interestingly, both of these previous works recognize that the observed coupling and uncoupling mediated by the varying GA regimes could have a close relationship with the transcriptional activity of DELLA proteins, known as important repressors of GA signaling.

RKN-soybean microarray studies have also consistently showed the key role of differences in ROS concentrations, defense genes and inductions of toxins in the resistance mechanism during pathogen attack [14,15]. However, few or none of them have focused on aspects related to the role of the hormone in biosynthetic processes and signaling pathways as mediators of resistance reaction.

Here in we describe the use of pyrosequencing to perform a comprehensive gene expression profiling of the soybean PI 595099–RKN pathosystem, leading to novel insights into incompatible interactions. We discuss the role of the plant hormones in biosynthetic processes and defense signaling against nematode invasion and suggest the modulation of ROS levels by auxin and gibberellin interactions, including new findings regarding the participation of DELLA-like proteins possibly controlling plant immune and stress responses.

## Results

### Time course analyses of RKN infection and development in compatible and incompatible interactions

**Figure 1:**
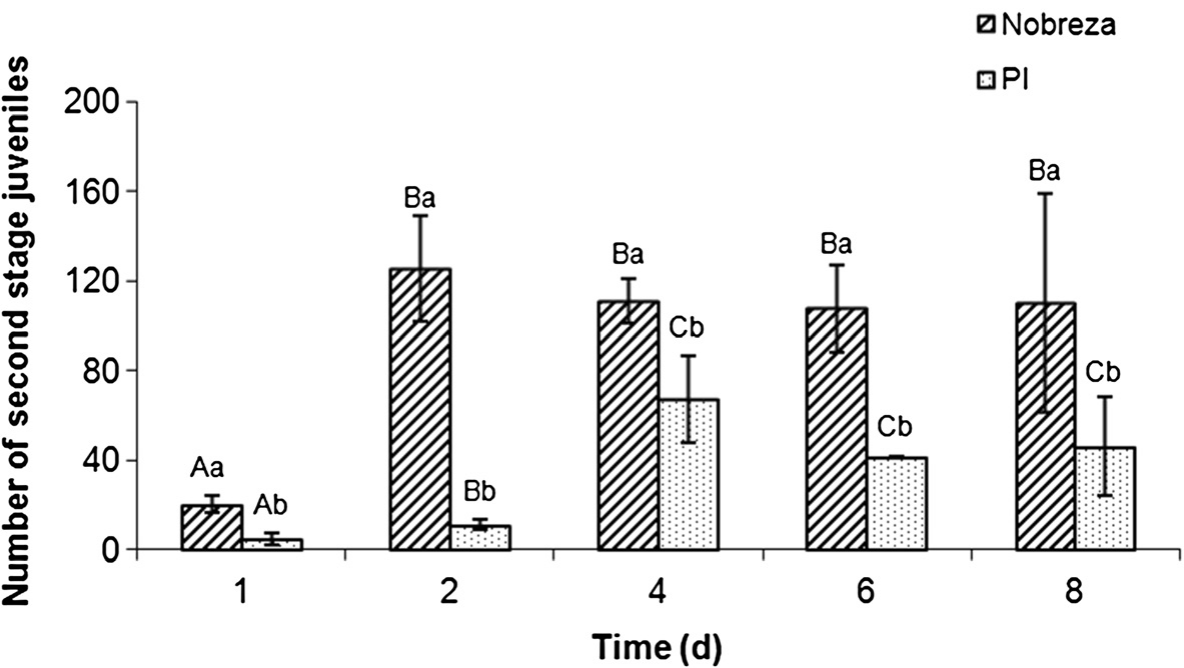
**Infection response of *M. javanica *in soybean roots.** Number of *M. javanica* J_2_ in soybean roots of susceptible (Nobreza) and resistant (PI 595099) genotypes at 1, 2, 4, 6 and 8 DAI. Capital letters represent the time courses within each genotype and small letters represent the comparison between cultivars in each time course. Bars followed by the same letter do not differ significantly at P ≤ 0.05 according to Poisson distribution method.

**Figure 2:**
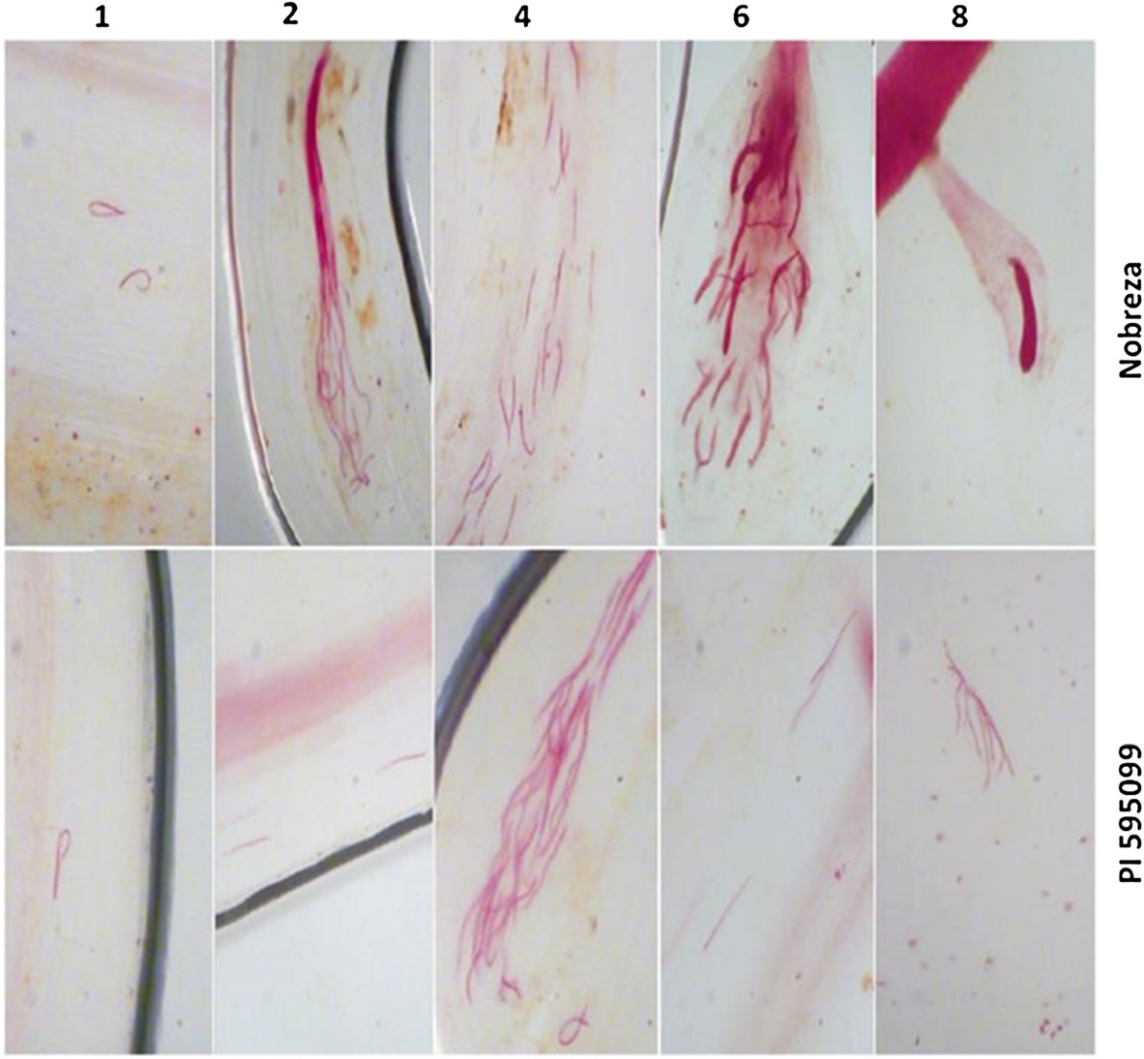
**Penetration of *M. javanica *in soybean roots.** Micro pictures of *M. javanica* J_2_ in soybean roots from Nobreza and PI 595099 at 1, 2, 4, 6 and 8 DAI. J3/J4 stages were observed only in ‘Nobreza’.

The analysis indicated that *M. javanica* juveniles invaded the soybean roots of both genotypes: Nobreza and PI 595099 (Figure 1). However, the maximum number of infective stage J_2_ differed significantly for both genotypes in all five experimental conditions: 1, 2, 4, 6 and 8 days after infection (DAI) from which root samples were collected for fuchsin staining. Statistical analysis displayed significant higher J_2_ infestation in the case of susceptible genotype Nobreza in the interval of 2 to 8 DAI. In the resistant genotype PI 595099 the highest J_2_ infestation occurred on the fourth DAI, although it did not represent a significant difference when compared to 6 and 8 DAI. Besides, in the PI 595099 the majority of juveniles were filiform, porous and less-densely stained at 6 DAI. At 8 DAI most of nematodes which penetrate in the susceptible soybean roots developed into J3/J4 stages (Figure 2).

**Figure 3:**
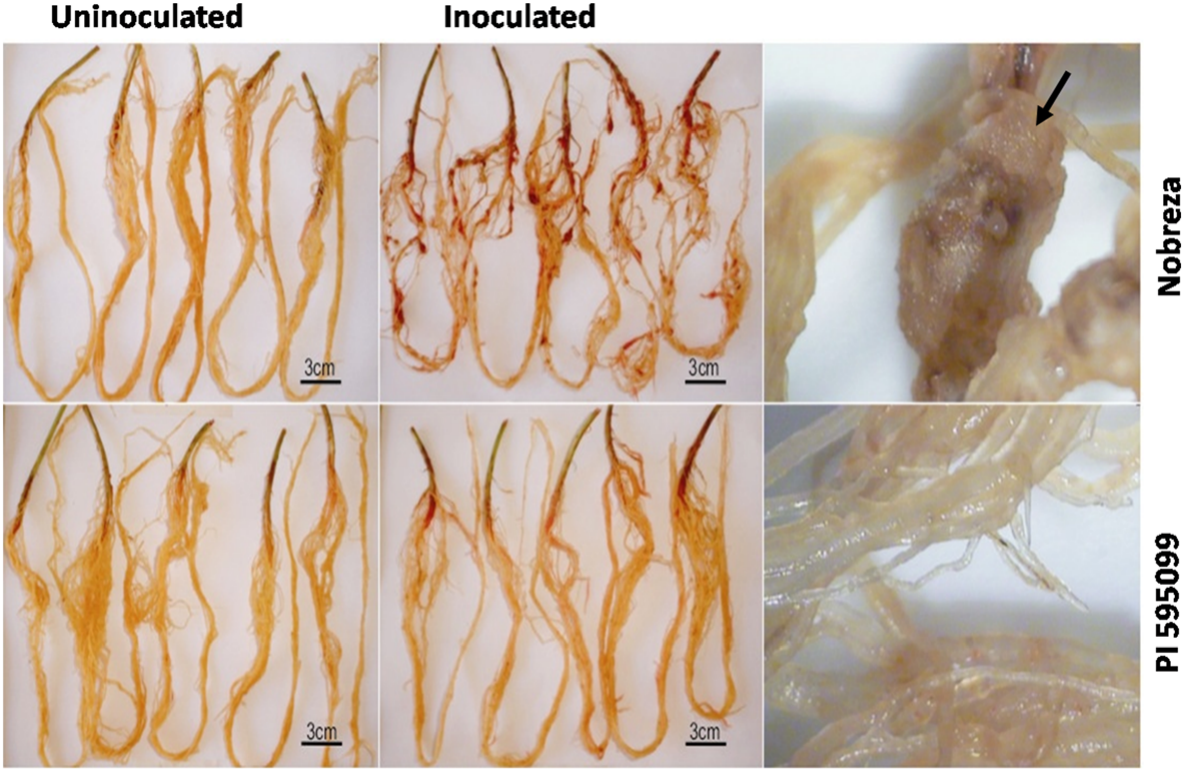
**Formation of galls in the soybean roots at 45 DAI.** Micro pictures show roots from soybean genotypes Nobreza and PI 595099 in the two treatments (mock-inoculated and inoculated) with *M. javanica* J_2_. The black arrow indicates the presence of galls in the roots of susceptible genotype (Nobreza).

The nematode’s ability to invade roots, measured by the number of galls and egg masses, as well as the total population of eggs and juveniles, is shown in Additional files 1 and 2, respectively. Significant differences were observed for both genotypes. ‘Nobreza’ showed a higher number of galls and egg masses per root milligram (Additional file 1) and, consequently, a higher number of eggs and juveniles (Additional file 2). In Figure 3 the result for the formation of galls at 45 DAI is presented, comparing two experimental conditions (mock-inoculated and PI 595099-inoculated). The presence of galls was found only in the susceptible genotype.

### Transcript assembly

A total of 1,348,738 expressed sequence tags (ESTs) with an average size of 350 bp were generated by 454-pyrosequencing in a single run. The Est2assembly pipeline [16] was used in a primary filtering step to produce 1,225,621 reads subjected to sequence assembly using a genome-guided approach taking advantage of the availability of the Soybean reference genome (Phytozome, Glyma1.0). Processing using PASA [17] software resulted in the validation of 1,147,865 ESTs alignment. The remaining non-aligned - EST subset was converted to Fastq sequence format [18] using biopieces bioinformatic framework utilities (http://code.google.com/p/biopieces/) and was used as input for a *de novo* transcript assembly with Mira Assembler version 3.4.0 [19]. Resulting sequence clusters were then used to feed another PASA run providing a new dataset of cluster alignments. The valid EST alignments and cluster alignments were then grouped based on genome mapping location and assembled into gene structures that include the maximum number of compatible transcript alignments.

PASA resulting clusters that mapped to the same genomic locus with a significant overlap and transcribed on the same strand were finally grouped into clusters of assemblies using bedtools software utilities (http://code.google.com/p/bedtools/), which yielded 102,871 clusters encompassing 1,148,451 ESTs (674,089 from inoculated sample and 474,362 from the mock-inoculated sample). Gene model refinement was carried out based on plausible open–reading frame lengths, resulting in 37,707 predicted gene models. Additional file 3 summarizes findings in the various transcriptome assembly steps.

### Gene expression analysis in the incompatible soybean (PI 595099)-RKN interaction

Transcript quantification for each sample was conducted using in-house custom perl scripts developed to extract the number of reads aligned to each one of the 37,707 assigned gene models. To leverage the power of the downstream analysis, clusters that matched gene predictions with a low number of members (< 5 sequences) were not included for differential expression analysis.

Differential expression was quantified using the software Glm edgeR to gene counts model fitting and testing with glmFit and glmLRT procedures. Due to the absence of sequenced sample replicates and aware that our experiment has been pooled several treatment conditions it was not possible to immediately identify differentially expressed genes by pairwise comparisons between the sample groups. It is because that for the experiments suffering with these limitations it is not clear whether the genes at the significance statistical analysis are accurately measured by the methods implemented in the software that largely depends on reliable estimation of gene-specific biological variation or at least of the estimation of global biological variation across all genes [20].

**Figure 4:**
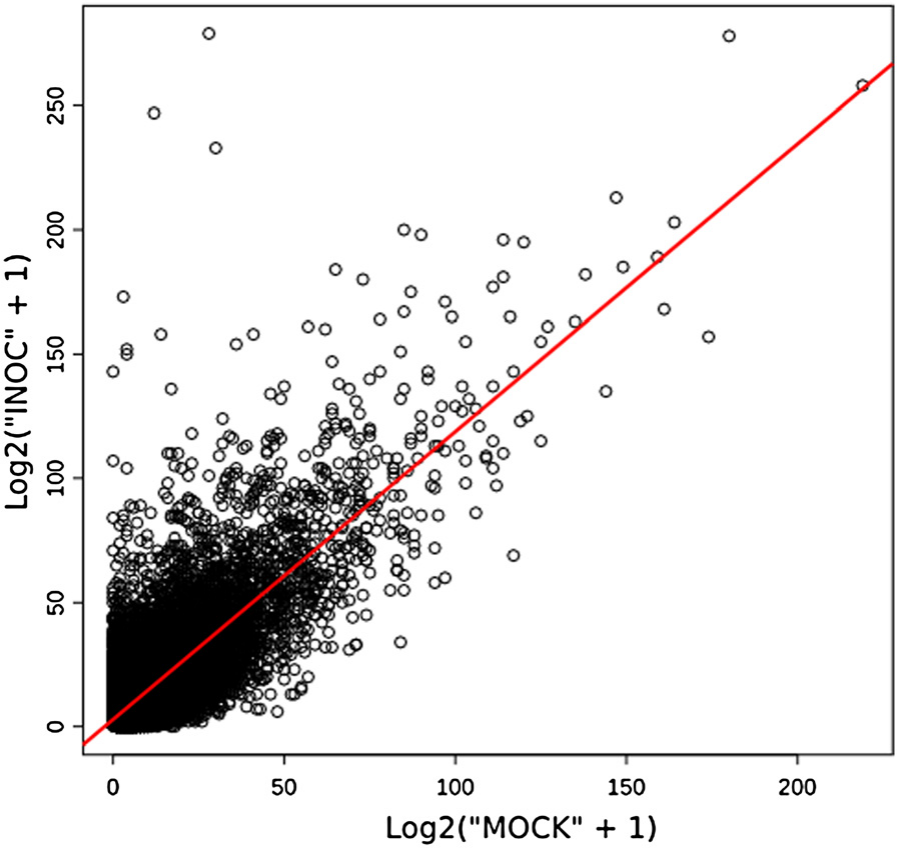
**Sample correlation for gene counts.** This plot determines the correlation level between gene counts of both mRNA samples sequenced in one single 454 run. Due to the wide range of expression data, a log2-transformation [log2(counts + 1)] is applied in order to improve the graphical representation. The Pearson’s correlation coefficient which indicates if both samples present a linear relationship is 0.80. Qualimap [78] R routines were used to draw the plot.

The descriptive analysis of the sequence data shows that the gene counts are highly correlated across samples (average Pearson correlation = 0.80) (Figure 4). Albeit a rough indication that sequence data is replicable it points up to a reduced effect from the two samples being sequenced at same concentrations in the separated gaskets of one picotiter plate. The distribution of the relative gene count abundance was accounted as the logarithm of the fold-change expression between the two samples after choosing a nominal average dispersion value of 0.25 (BCV = 50%) and proceed to gene counts model fitting with edgeR. While we are not undoubtedly de-prioritizing genes with inconsistent measures of statistical significance with this nominal choice of average sequence data dispersion, it should allow us to focus on changes that could be consistent between replicates were the observed BCV is typical of what is reported in mRNA-Seq studies with next-generation sequencing data [20].

To isolate a subset of genes possibly related to the host incompatible reaction to the nematode invasion we initially adopted a lenient analysis of the pairwise sample comparison in terms of P-values and FDRs. At raw P-values < 0.1, a comparison of the samples yields 3,933 differentially expressed genes while the P-values adjusted to control the false discovery rate (FDR) by the method of Benjamini and Hochberg ranged from 0.08 up to 0.66, potentially resulting in a large number of false discoveries.

A second step in our pipeline relied in the ranking-test of the calculated fold-change expression for that subset of genes to detect enrichment of particular biological processes in the inoculated sample described in terms of Gene Ontologies (GO). This allowed us to determine whether the rank for a particular GO was significantly higher or lower than usual and accordingly summarizes the result as a differential gene list into various GO categories (Additional file 4). Following this approach solely based on bioinformatics analysis we detect 725 genes possibly involved in the plant responses to the nematode invasion. From these 586 genes were up-regulated in the nematode-infected roots, whereas 139 were down-regulated when compared to the mock-inoculated control. These observations point up to a significant imbalance of gene expression after nematode infection.

### Association of expressed genes in functional categories using Gene Ontologies (GO)

*In silico* analyses for detecting significant GO associations were performed using a Wilcoxon Rank test [21]. The convention was to use the mock-inoculated root sample as a background for gene counts, meaning that the continuous variable logarithm of the fold-change expression represents the ranked probability score for the gene being not differentially expressed. In this way, the lower the signed value, the more likely that gene is being not differentially expressed. By specifying the ranked probability, we could roughly capture the transcriptional changes by noting an increase in the ranking for particular GO terms representing oxidation-reduction process, response to different stimulus, carbohydrate metabolism, hormone biosynthesis and signaling (Table 1).

**Table 1:** GO statistical analysis for analyzing transcript sequences

**Node**	**Term**	**Genes outside node**	**Genes in node**	**Significant**	**p value**
GO:0055114	Oxidation-reduction process	1085	116	108	8.26E-005
GO:0006979	Response to oxidative stress	1127	74	63	0.0015
GO:0005975	Carbohydrate metabolic process	1090	111	61	0.0804
GO:0042538	Hyperosmotic salinity response	1184	17	17	0.0008
GO:0009414	Response to water deprivation	1172	29	13	0.0049
GO:0007010	Cytoskeleton organization	1148	53	8	0.0032
GO:0009813	Flavonoid biosynthetic process	1177	24	7	0.0029
GO:0009807	Lignan biosynthetic process	1195	6	6	0.0133
GO:0009266	Response to temperature stimulus	1122	79	6	0.0406
GO:0046274	Lignin catabolic process	1195	6	6	0.0675
GO:0009718	Anthocyanin biosynthetic process	1191	10	5	0.0006
GO:0009627	Systemic acquired resistance	1194	7	5	0.0073
GO:0000165	MAPK cascade	1196	5	5	0.0091
GO:0009741	Response to brassinosteroid stimulus	1195	6	5	0.0302
GO:0009699	Phenylpropanoid biosynthetic process	1165	36	3	9.85E-005
GO:0009850	Auxin metabolic process	1196	5	3	0.0154
GO:0000272	Polysaccharide catabolic process	1181	20	3	0.0485
GO:0009698	Phenylpropanoid metabolic process	1155	46	2	9.93E-005
GO:0009808	Lignin metabolic process	1185	16	2	0.01864
GO:0009938	Negative regulation of gibberellic acid mediated signaling pathway	1199	2	2	0.0624
GO:0009851	Auxin biosynthetic process	1199	2	1	0.0154
GO:0009861	Jasmonic acid and ethylene-dependent systemic resistance	1200	1	1	0.0749

Gene Ontology (GO) terms identified to be enriched or depleted according to the comparison of the distribution of the gene-associated Log2FC among all differentially expressed genes classified in the GO category “*biological process*”. The value of Log2FC obtained by differential expression analysis (at p-value < =0.1) was used as the measure of choice to rank genes as “differentially expressed” or “not differentially expressed”. Then a Wilcoxon-rank test was performed with 1201 genes to compare the ranks of the genes in the tested GO term (Genes in node) with the ranks of the remaining genes in the top category (Genes not in node). For each comparison a p-value was obtained and a cut-off provided as the global test statistic to gauge the significance of the complete data set was used to establish the number of genes with relevant associations with each tested category (Significant).

**Figure 5:**
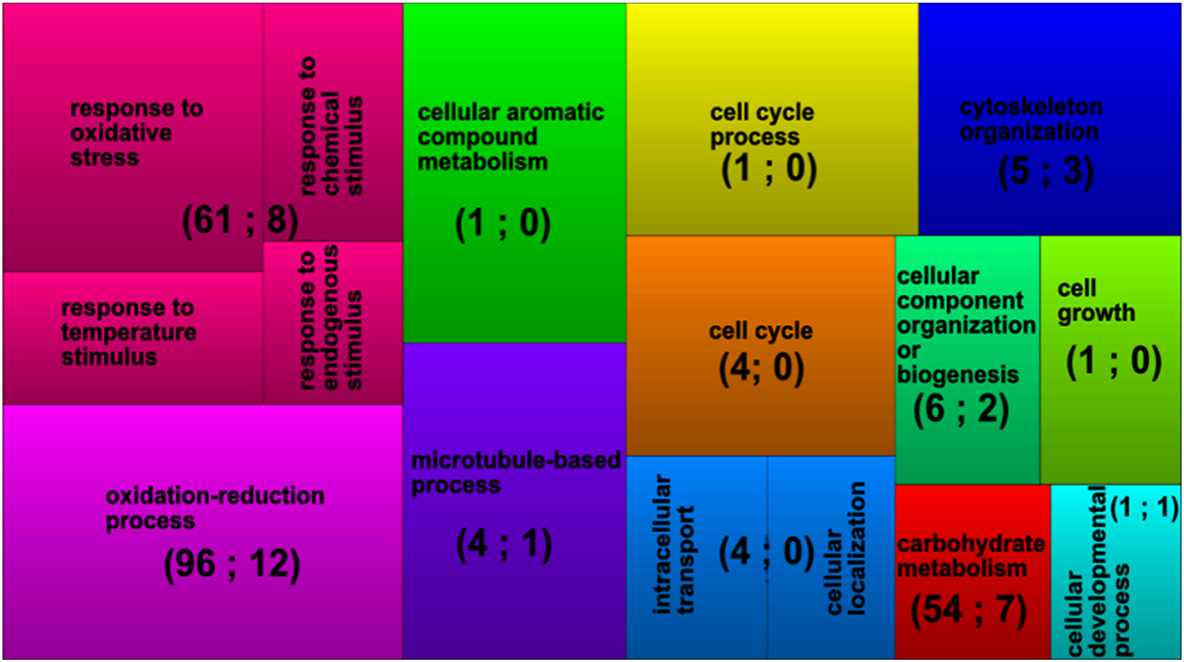
**Plot summaries for the GO enrichment analysis.** GO terms were identified to be enriched according to the comparison of gene-associated Log2FC distribution among all differentially expressed genes classified in the category “*biological process*”. Each rectangle is a single cluster term representative while a “supercluster” of loosely related terms is visualized with different colors. Rectangles are size–coded to reflect the frequency of the GO term in the underlying AgriGo soybean reference dataset. Numbers inside of parentheses represent the number of genes up- and down-regulated at statistical significance level for the differential expression analysis (p-value < 0.1) taken the mock-inoculated sample as the control.

In order to summarize the differential gene list into various GO categories, terms showing higher ranking by the Wilcoxon Rank statistical test were embedded in a two–dimensional space using the software REViGO [22]. The resulting treemap-plots capture the inter–relations of the most significant GO terms associations belonging to “biological process” (Figure 5). As expected from earlier Rank test the biological process ontology category is dominated by activities related to redox reactions. Other prominent processes were a response to chemical, endogenous, temperature and oxidative stress and carbohydrate metabolism. Considering the analyses concerning gene expression level, we noted that up-regulated genes included those encoding glycosyltransferases, peroxidases, auxin and gibberellin-regulated genes (Additional file 4).

### Biological functions of representative genes by domain analysis

**Figure 6:**
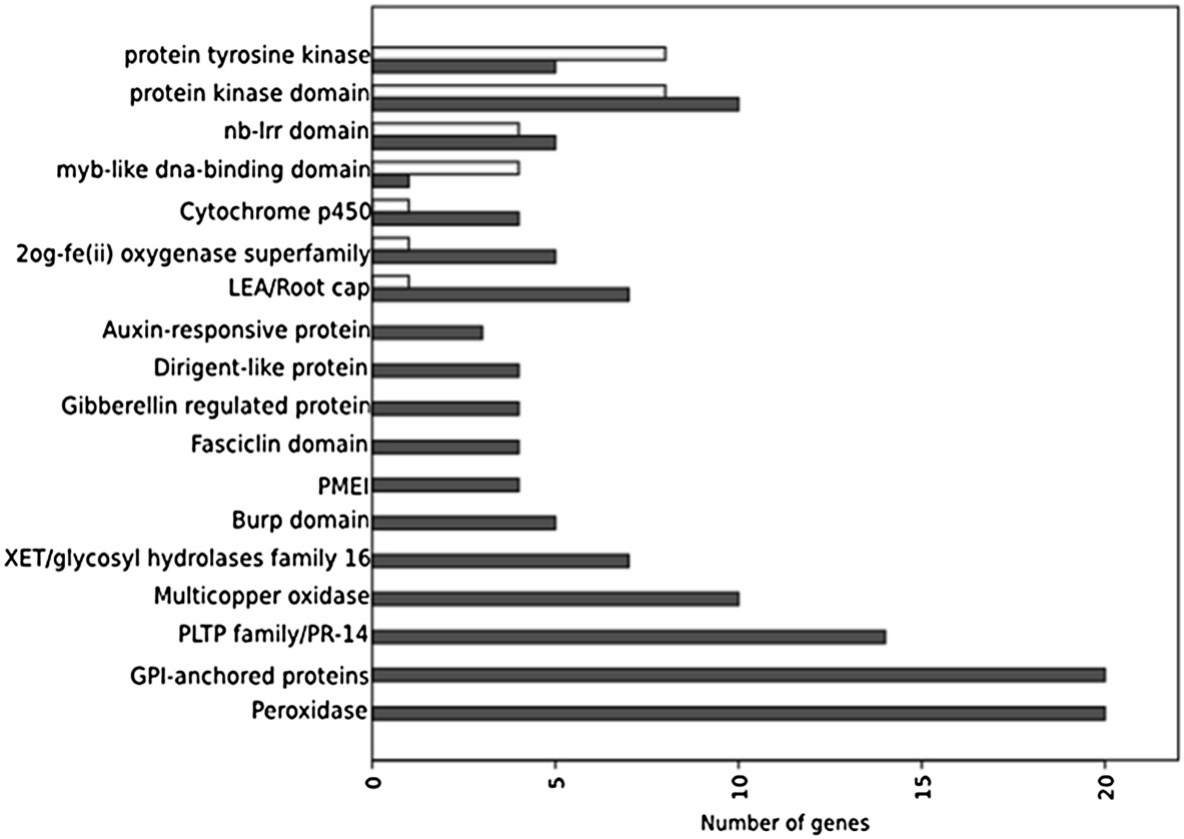
**Pfam domain frequency distribution for the differentially expressed genes.** The corresponding numbers of up–regulated genes in the infected sample are shown (dark grey bars). Genes in the mock-inoculated sample are also represented whenever available (bright grey bars).

Co-expressed genes may contain structurally similar protein motifs [23] and therefore, we ran a domain analysis on the sequences in the achieved gene list. The most common domains found are shown in Figure 6. It is possible to verify that some gene sets contain particular domain families that are otherwise under-represented or not represented at all in the control. In the inoculated root sample the most striking observation is the occurrence of domains present in proteins involved in oxidation–reduction reactions, particularly multicopper oxidase (PF00394, PF07731 and PF07732) and oxoglutarate/iron-dependent dioxygenase (PF03171) domain families; as well as domains involved in response to oxidative stress with peroxidase (PF00141) being the most prevalent domain. Other noticeable domains include those in proteins from genes related to carbohydrate and cell wall metabolism, including pectin methylesterases and their inhibitors (PMEI) (PF04043 and PF01095) and O-Glycosyl hydrolases (PF00722, PF00933 and PF01915).

We also find domains that suggest a role in plant defense or stress responses that are over-expressed in the inoculated root sample. These include gene sequences containing the Plant Lipid Transfer Proteins (PLTPs) domain, which are also referred as plant Pathogenesis-Related (PR-14) proteins, BURP domain proteins and Fasciclin-Like Arabinogalactan-proteins (FLA) (PF02469). It was also detected significant induction in genes encoding proteins that are responsive to the hormone auxin: containing Auxin-inducible, GH3 and AUX_IAA domains and proteins regulated by the hormone plant gibberellins. Over-expression of genes coding for the APETALA2 transcription factors family, EREBP (Ethylene-responsive element binding protein) and Leafy Petiole (LEP) were also noted (Additional file 4A).

### Biological relevance of detected genes

To demonstrate further the biological relevance of the detected genes tested for Gene Ontology enrichment association with representative biological processes we looked for a previous work [24] where genetic markers were found in significance statistical association with QTLs conditioning PI 595099 resistance against *M. javanica*. In that study, crosses between PI 595099 genotype and BRS 133 (soybean susceptible genotype to *M. javanica* infestation) were investigated by twenty-one polymorphic SSR markers where seven markers showed significantly different in the resistant population. From these, at least three SSR loci, SOYHSP176, Satt114 and Satt571, showed a significant correlation with the number of galls observed on infected soybean roots.

Genomic locations of the seven Soybean SSR markers (Satt114, Satt571, Satt419, Satt367, Sat_128, Sat_132 and SOYHSP176) were obtained from Soybase (http://www.soybase.org). We looked in the tested gene dataset for enrichment for those genes which genomic coordinates were within a window of up to 5 cM (centimorgan) from each of the SSR markers. For this purpose a naive conversion from genetic distance to base pairs distance was adopted using the estimated size of Soybean genome (975 Mb) (http://www.phytozome.net/soybean.php) and the Soybean genetic map length (~2500 cM) [25] resulting 1 cM ~ (975 M/2500) ~ 390 Kb.

Within the 5 cM window from SSR loci we found that six of the seven markers (Sat_128, Satt114, Satt367, Satt419, Satt571, SOYHSP176) overlaps the genomic coordinate of 72 genes encompassing transcriptional changes in our pipeline as measured by the fold changes and rank-test. Those genes mainly encode proteins involved in oxidation-reduction reactions as multicopper oxidase, peroxidases and NADPH thioredoxin reductase (TR). Vesicle-mediate transport proteins as from the SNARE complex/VAMP superfamily, gibberellin 2-oxidase, calcium-dependent and calmodulin related gene encoding proteins as well as genes functioning as glycosyltransferase, O-glycosyl hydrolase and pectinesterase activity were also observed in this subset (Additional file 4B). Interestingly, the markers Satt114 and SOYHSP176 encompassing the greater number of genes within the 5 cM window (37 genes) showed the most significance statistical association with the reduced number of galls observed on infected resistant soybean roots [24].

### Transcriptome of PI 595099 encompasses a great diversity of gene families as important players in the parasitic biotrophic-root interaction

Given the diversity of gene families possibly found to undergo transcriptional changes as pointed up in the previous results we believe that the parasitic biotroph-root interaction can greatly rely on networks of interconnected signaling pathways into the plant host. Multiple hormonal signals, mainly auxins, gibberellins and brassinosteroids as well ethylene and jasmonates, are possibly involved in the coordinated regulation of biological processes that cope with the stress responses and defense reactions against RKNs.

**Figure 7:**
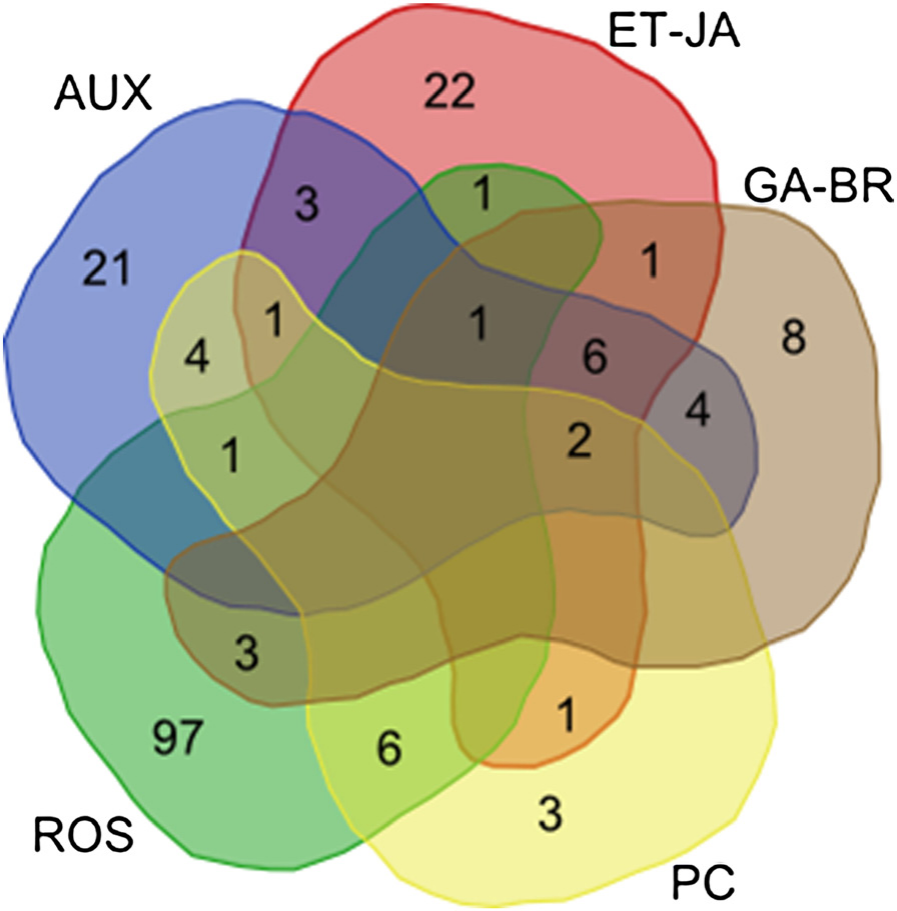
**Venn diagram for differentially expressed genes involved in RKN resistance in PI 595099.** The numbers in the graph represent the number of genes differentially expressed as measured by differential expression analysis (p-value < = 0.1). Gene sets were grouped according to GO annotation obtained from the agriGO database for the gene ontology analysis and Phytozome (see Methods and Table S3). AUX, GA-BR, ET-JA include genes annotated in biological processes that in which plant hormones auxins, gibberellins or brassinosteroids and ethylene or jasmonates participate, respectively. ROS include genes annotated in the oxidation-reduction and response to oxidative stress GO terms. “Phenolic compounds (PC)” include genes annotated in biological processes related to biosynthesis pathways leading to the formation of main groups of phenolic compounds (phenylopropanoids and flavonoids). The search was performed on the GO associations listed in Table S3 using the key words “auxin*”, “gibberell*” OR brassinosteroid*”, “ethylene OR jasmon*”, “oxidation-reduction OR oxidative” and “flavon* OR phenylprop*”.

**Figure 8:**
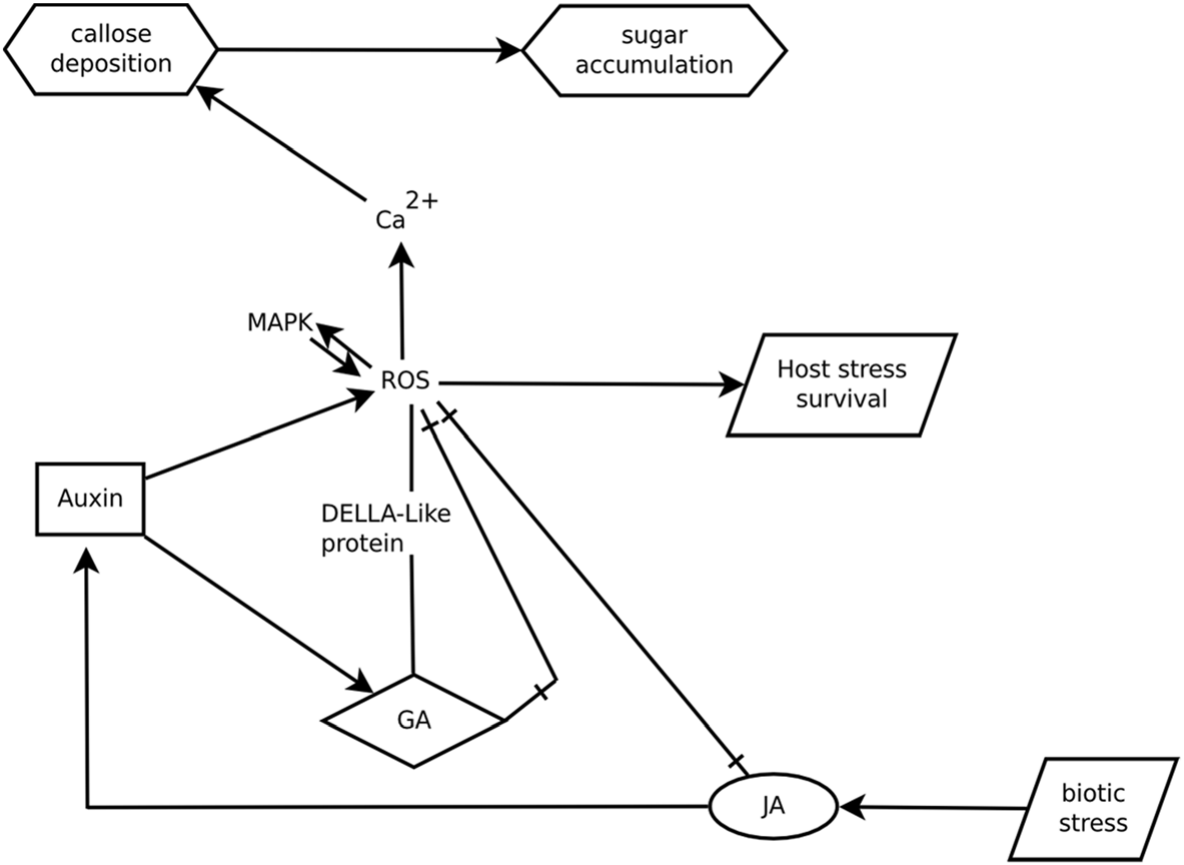
**Plausible model for gene regulation in RKN resistance in PI 595099 soybean line.** This attempted model is largely based on diagram shown in Mitler et al. (2011) about the integration of reactive oxygen species (ROS) and hormonal signaling networks and stresses the importance of ROS in controlling plant immunity and stress responses. Elevation of Ca^2+^ levels and activity of nucleotide sugar forming enzymes as sucrose synthase and glycosytransferases through ROS mediated signaling is proposed to have a critical role in controlling the changes in endogenous hormone levels that lead to adaptive responses of the plant host after nematode invasion. Among these responses are the activation of MAPK signaling pathways, fine-tuning modulating to the amplitude and onset of ROS levels, accumulation of anthocyanins and other phenolic compounds (PCs), as well as the activation of defense responses. Auxin-mediated defense mechanism is involved in resistance to the biotrophic nematode and largely depends on GA and JA signaling. Upon pathogen infection, endogenously elevated jasmonates (JA and MeJA) stimulates auxin biosynthesis while auxin enhances ROS signaled induction. Additionaly, jasmonates can induces the accumulation of ascorbate, glutathione and increases the activity of dehydroascorbate oxidase, involved in antioxidant activity. The amplitude and onset of ROS elevated levels in turn is accompanied by the induction of JAZ/TIFY class of repressors that contributes to prevent jasmonate biosynthesis. Elevated levels of auxin affect gibberellin biosynthesis leading to induction of gene expression levels of both GA biosynthetic and deactivating genes. Finally, a DELLA-like protein part of GA-pathway is induced upon pathogen attack and is proposed to act as a key element to integrate signals from auxin, GA and JA in both growth and defense response processes mainly causing ROS levels to remain low positively regulating anthocyanin accumulation and PCs as part of the redox system under stress conditions.

Recent findings [13,26] that demonstrate that hormonal signaling function as a mechanism appropriately regulating growth to adverse conditions, for example by the reduction in ROS levels, are in a good match to our observations on the gene expression profiling. Concerning this aspect, we carried out a search in the significant associations revealed by the previous bioinformatics analysis to generate a diagram showing the possible cross-interactions between biological process with involvement of plant hormones, reactive oxygen species and phenolic compounds. The resulting Venn diagram is shown in Figure 7, encompassing over 180 genes. Based on our findings we suggest a plausible model that is proposed to extend the view regarding the complex mechanisms of cross talk between gibberellin and other plant hormones, mainly auxins. This proposed model depicted in Figure 8 is discussed herein as a tentative model to include the new findings regarding the participation of DELLA-like proteins in plant immune or stress responses.

## Discussion

The soybean line PI 595099 presents high resistance to the main RKNs, as well as to *H. glycines*[27]. The low-level of galls and egg masses observed in PI 595099 in the present study indicates that this genotype has a resistance mechanism that limited RKN infection, which is consistent with preceding histological report [4,27,28]. Previous molecular studies using this soybean line showed differential expression of a set of genes when inoculated with *M. javanica* in comparison to the mock-inoculated [29,30]. However, they were not fully able to provide insights into the mechanisms operating in the host’s resistance. To provide an extensive characterization of the distinctive responses of PI 595099-RKN interaction we present large-scale transcript sequence data generated by NGS technology.

### PI 595099 comparative transcriptome analysis reveals complex stress signaling upon RKN infection

The gene expression profile analysis carried out using next generation sequencing data and bioinformatics analysis shows that PI595099 triggers a broad set of plant stress genes in response to nematode invasion between 0 to 8 DAI (Additional file 4). Our findings indicate that several members of Lipid transfer proteins (LTPs) family, also known as PR–14, which are important players in the general plant stress response, were induced during pathogen infection. Recently, it has been shown that LTPs are also involved in salt and drought stress [31], and have been implanted in Ca2+ signaling networks, due to the presence of a calmodulin binding region within this protein family [32]. This particular observation is in contrast to what was observed using microarrays to study the gene expression of a susceptible soybean cultivar and *M. incognita*[5], where PR–1, PR–2 and PR–5 protein families increased in expression, but not PR–14 members. Most noteworthy, the induction of expression of genes encoding PR-1 or plant defensin (PDF1.2), globally known as marker genes to study SA-dependent and ET/JA-mediated defense responses, did not take place in our analysis.

Another protein class that was identified include Arabinogalactan-proteins (AGPs), which contain a glycosylphosphatidylinositol-(GPI-) membrane anchor and have established roles in plant development and signaling [33], specifically cellulose deposition and cell wall plasticity [34]. Furthermore, AGPs were seen to be implicated in the production of mucilage during the incompatible interaction with parasitic plant [35] and their expression was found to increase in response to salt stress [36]. AGPs involvement in the protection against nematode infection has not previously been established and the observed up–regulation of AGPs can be the result of the plant’s efforts to recover from stress and resumption of its growth. Proteins containing the BURP domain (PF03181) were also significantly up–regulated. Although there is no precise functional characterization of this large family, recent reports corroborate over-expression following cyst nematode infection in tomato [37] and following abiotic stresses such as abscisic acid and NaCl treatment [38].

As LTPs, AGPs and BURP are thought to be related to salt stress tolerance, they may be involved in the common denominator of our findings named the increase of ROS production, which notably also occurs in situations like water deficit or salinity [39,40]. Furthermore, it has been shown that salt stress signaling shares several events with biotic defense in plants, including the formation of ROS and induction of JAZ/TIFY transcript levels, known as negative regulator of jasmonate signaling [41] also observed in the present study. Another interesting aspect concerning the suggestive overlapping between salt stress signaling and biotic defense in PI505999 may be the increase plant annexin abundance noted by the up-regulation of two genes putatively encoding members of this protein family (PF00191). Annexins abundance was already observed to increase in stress conditions like salinity, drought, metal stress, and exogenous abscisic acid and it was recently suggested that they could function in integrating ROS and Ca^2+^ in stress signaling [42].

The expression profiling analysis based on the associations between genes and their GO annotation also suggests a key role of glycosyltransferases, and gibberellin signal transduction proteins (incl. DELLA-like proteins, GA20ox and GA2ox) in the resistance reaction. In addition these results suggest the probable participation of these gene products in jasmonate signaling and redox homeostasis by mediating aspects of plant growth and biotic stress responses (Additional file 4). It clearly reinforces the recent findings demonstrating that hormonal signaling can also function as a mechanism regulating growth to adverse conditions, for example by the reduction in ROS levels [13,26].

### ROS generation as a defense against nematode infection

Activation of various oxidases and peroxidases in response to adverse environmental factors led to the production of ROS, comprising superoxide anion radical, singlet oxygen, hydrogen peroxide, and hydroxyl radicals. Respiratory burst oxidases (NADPH-dependent oxidases) are thought to be important sources of ROS in response to pathogen attack [43]. A total of 17 members of this family were detected without sign of expression modulation. NADPH-oxidases activity has been also attributed to apoplastic peroxidases in some plant species challenged by pathogens and after elicitor treatment. Peroxibase [44] reports 19 records of soybean peroxidases with homology to respiratory burst oxidases but none of them were found with a sign of expression modulation in our study.

Alternatively, increased levels of ROS may also be a consequence of the action of plant hormones, altered sugar levels and fatty acids [45]. Auxins were hypothesized to induce cell-specific ROS formation and affect cell antioxidant content in response to environmentally unfavorable conditions. Accordingly, cells of the quiescent centre can accumulate high auxin levels and contribute to the overproduction of ROS affecting the ROS/antioxidant balance in the root apical meristem. This mechanism of action comprises auxin binding to the TIR1 receptor leading to removal of transcriptional repression of a large array of genes possessing the Auxin Response Factor (ARF) signature and causing the generation of H_2_O_2_ and superoxide ions [46].

Apparently, high levels of auxins, mainly in the form of free indole-3-acetic acid (IAA), can arise in the interplay between IAA biosynthesis and conjugation in the maintenance of IAA gradients [8]. In the inoculated sample we found an over-expressed gene (*GH3.6*) which the encoded enzyme functions in the synthesis of IAA-amino acid conjugates. Accordingly, the over-expression of *GH3* might lead to the accumulation of IAA-Asp conjugates which is a potential mechanism for plant cells to cope with presence of auxin excess in its bioactive form [47]. This *GH3*-mediated auxin homeostasis also was proposed as an essential constituent of auxin actions that regulate stress adaptation responses in plants [48]. Furthermore, our study also found a number of up-regulated genes, encoding family domains associated with induction by the plant hormone auxin, such as AUX/IAA and small auxin-up RNA (SAUR).

Jasmonic acid (JA) and its methyl ester (methyl jasmonate, MeJa) were reported to enhance the production of ROS, especially H_2_0_2_, and to induce cell death synergistically with other plant hormones such as ethylene and salicylic acid [49]. It also has been suggested that the amplitude and onset of elevated ROS levels in tolerant or resistant plant genotypes might behave antagonistically to events such as induction of JAZ/TIFY transcripts (jasmonate ZIM/tify-domain) and apoplastic alkalinization [41]. In this scenario, jasmonate action might correlate with adaptive responses that modulate ROS accumulation instead of promoting the accumulation of ROS that could lead to cellular damage. This latter interpretation of jasmonate’s action as part of a defense reaction seems also to occur in the studied pathosystem, since we could observe enhanced activity of one gene coding for allene oxide cyclase (AOC) class of enzymes in the inoculated sample, which acts in the primary steps of jasmonate biosynthesis, and also observed the induction of two genes encoding JAZ/TIFY proteins. It is also noteworthy in our study that the production of jasmonates might be rate limited by the down-regulation of the acy-CoA oxidase (ACX) gene, which gene product is one of the core enzymes that catalyze the final steps of JA synthesis. ACX is also known as an H_2_0_2_-generating enzyme that acts into the peroxisome.

In parallel, an enhancement was observed of the activity of the enzyme ascorbate (ASC) oxidase, which is known to oxidize ascorbate to dehydroascorbate (DHA), a molecule able to directly interact with reduced glutathione (GSH) and thiol-containing proteins. ASC are potentially important components regulating redox-sensitive proteins via auxin functioning. Between the up-regulated genes containing multicopper oxidase domains, we observed two genes encoding the enzyme ASC oxidase (EC 1.10.3.3). Thiol-containing proteins were also found up-regulated in the infected sample at significant expression levels.

### Antioxidant status as part of defense against nematode infection and signaling pathways

Given the over–representation of genes containing various oxidases and peroxidases domains in the infected sample, it was done a more detailed analysis of transcripts related to ROS antioxidant activity since the ROS release may in turn induce ROS scavengers and other protective mechanisms.

Peroxidases are key player in the detoxification of reactive oxygen species during cellular metabolism and oxidative stress. The annotation of all up–regulated genes containing the peroxidase domain reveals that they were found to belong to class III plant peroxidases (EC 1.11.1.7). This class is known to participate in many different plant processes, such as auxin metabolism, cell wall elongation and stiffening and protection against pathogens [50]. Recently it was demonstrated that these class III peroxidases are located at the tonoplast and plasma membrane and are able to catalyze the reduction of hydrogen peroxide by taking electrons to various donor molecules, such as phenolic compounds, lignin precursors, auxin or secondary metabolites [51].

Other genes encoding enzymes with known antioxidant activity, such as catalases and ascorbate peroxidases were found in our survey, but without sign of expression modulation. Only one germin-like protein coding gene with putative superoxide dismutase (SOD) activity was found up-regulated in the infected sample, as well only one gene coding for glutathione peroxidase enzyme. Up-regulation of gene expression of SNARE complex proteins and SYP111, SYP121 and SYP132 members of SYP1 of plasma membrane-localized syntaxins family [52] was found in our study. This suggest that further investigations on the intracellular localization of ROS is needed to elucidate whether membrane trafficking is an important characteristic in cellular responses to nematode invasion.

Our study also showed that transcriptional activity of glutathione S-transferases (GST) genes increased in the inoculated sample, as well as many PR protein encoding genes, mainly group 5 (PR-5; Pfam: PF00314) and LTPs (PR-14; Pfam: PF00234), and a phenylalanine ammonia-lyase (PAL) gene (Pfam:PF00221; EC:4.3.1.24). GST is known to function in hydroperoxide detoxification through reduction of peroxides with the help of GSH and PR. PAL proteins were identified as inhibitors of H_2_O_2_ production [53]. Taken together, these observations may suggest that the host plants have the ability to adapt very well to low activities of both catalase and ascorbate peroxidase by induction of other defense systems, probably by signaled induction.

Increasing evidence indicates that ROS function in plants also as signaling molecules involved in regulating development and pathogen defense responses [53,54]. Pathways of ROS signaling are reported to participate in homeostatic regulation by antioxidant redox buffering, which provides robust protection against oxidative stress. The ability of GSH to act as redox buffer is one of the most important attributes of plant cells. In the cytoplasm, signaling linked to increased availability of ROS may be caused, limited, or mediated by changes in the redox buffering capacity. Therefore such way, any stimulus that perturbs cellular redox balance may serve as an inducer for a set of defense-related genes, including PR proteins. Key redox signaling components are thioredoxins (TRX) and glutaredoxins (GRX), which are reduced by ferredoxin, NADPH thioredoxin reductase (TR), or glutathione [55]. Members of thiol-containing families were found at increased expression levels in the infected samples and may have an important role in the redox signal transduction. Another interesting finding is the significant up-regulation of one gene encoding an NADPH thioredoxin reductase (TR) enzyme (EC: 1.8.1.9). Recently, it was found that purified TRX can reduce oxidized glutathione (GSSG) to GSH in the presence of TR and NADPH in a reconstituted *in vitro* system in yeast [56].

Unlike the cytoplasm, the apoplast is deficient in glutathione and therefore its redox buffering capacity is considered weaker. However, the apoplast is considered crucial in facilitating ROS-mediated signaling by maintaining a balance of reduced and oxidized forms of ascorbate (ASC). This ascorbate-based system is reported as important in driving plasma membrane and tonoplast electron transport chains by influencing cell wall composition. Moreover, low apoplastic antioxidant buffering capacity establishes a steep redox gradient across both the plasma membrane and the tonoplast. Low buffering capacity also permits further reactions to be triggered by secondary oxidant-induced signaling events in the cell wall, such as release of small oligosaccharides that are generated during the breakdown of pectins (pectic polysaccharides) [55]. Many differentially expressed genes encoding transferase enzymes (PF02458) involved in the biosynthesis of soluble phenolic compounds (PCs) were found, which were that accumulated in the inoculated sample.

A PAL gene found under up-regulation in our study encodes a key enzyme for the biosynthesis of anthocyanin and other PCs. Although it is widely recognized that PCs are involved in the H_2_O_2_ scavenging cascade in plant cells [57], it was only recently that their accumulation was postulated to form part of an integrated redox system, quenching ROS and contributing to stress tolerance [45,58]. The model proposed to integrate PCs into a redox system is supposed to largely depend on cellular nucleotide sugar concentrations. Nucleotide sugars linked to a nucleotide-diphosphate (NDP-sugars) can serve as donor substrates for glycosyltransferases (GT) that transfer sugar to a wide range of acceptors and can directly affect bioactivity of diverse plant hormones, as well as defense-related small molecules [59]. Transcriptomic analysis [60] revealed a number of potential GT transcripts up-regulated in response to methyl jasmonate, and their co-expression relative to that of β-amyrin synthase.

### Gibberellin and auxin act as key in integrating ROS signaling pathways during (PI 595099)-RKN interaction

The phytohormone gibberellin (GA) and its signaling components have been shown to play important roles in plant defense [61,62]. However, little is known about their effects on metabolic adjustments and influence on the fine-tuning ROS levels in response to plant stress. In view of this, H_2_O_2_ was observed to be implicated in activation of GA synthesis and signaling [63]. Furthermore, it has been shown that variation in GA levels can provide a mechanism for plant growth regulation, causing ROS levels to remain low after biotic or abiotic stress [13].

Investigations of GA regulatory mechanism in plants under salt and mannitol stress and pathogen interaction led to identification of a large set of differentially expressed genes DELLA-dependent. Interestingly, a wide range of those genes was found to be responsive to oxidative stress, encoding known antioxidant systems such as SOD, peroxidases or GSTs [13]. Thus, it was suggested that DELLA proteins can accumulate under stress conditions through reduction in GA levels and in turn activate a complex genetic regulation network to control ROS. Additionally, DELLA proteins can positively regulate anthocyanin accumulation related to nutrient stress [64], providing another link between GA-DELLA and ROS regulation.

Auxin was previously thought to interact positively with gibberellin to promote GA responses by destabilizing DELLA and by inducing the expression of GA biosynthetic genes, such as GA20ox and GA3ox, and leading to the down-regulation of GA catabolism genes such as GA2ox [65]. This interaction was proposed to occur through a DELLA-independent pathway by the removal of transcriptional repression of a large array of genes possessing the Auxin Response Factor (ARF) signature through the degradation of auxin signaling suppressors Aux/IAA proteins. Therefore, the effect of auxins, such as indole-3-acetic acid (IAA), is thought to be at least in part mediated by its effect on GA metabolism [66]. However, under adverse environmental conditions, proteins which belong to a subfamily of the GRAS protein can accumulate in the cell and function as repressors of GA signaling playing a prominent role in the auxin-gibberellin interplay through the maintenance of reduced levels of gibberellic acids [67].

One interesting protein found in our study regards great similarity to Glyma05g03490, a gene that encodes a protein containing GRASS domain, and exhibits high expression level in the inoculated sample. Similarity analysis using tblastn program indicates that the Glyma05g03490 gene shares suggestive homology (55% identity and 70% similarity) with the rice locus Os06g0127800 gene encoded protein. Further investigation accounts that the Glyma05g03490 encoded protein shares GRAS domains such as LHRI, VHIID, LHR, PFYRE and SAW motifs with the Os06g0127800 encoded protein, but both of them lack DELLA and TVHYNP motifs found conserved in DELLA proteins. Recently, the locus Os06g0127800 was amplified from *dwarf 62(d62)* rice mutant inducing the dwarf phenotype with increased gene expression levels of both GA biosynthetic and deactivating genes, OsGA20ox2 and OsGA2ox3, respectively [68].

In our study, the scenario described above depicts a plausible interpretation of the observed expression patterns on the possible interactions between auxin, gibberellin, jasmonate and ROS related responses. As illustrated in the Figure 8 this model involves the participation of auxins, DELLA-like (d62 rice gene related) proteins and JAZ/TIFFY proteins in controlling plant immunity and stress responses through the modulation of the amplitude and onset of elevated ROS levels. Significative findings supporting this view can include the up-regulation of GA biosynthetic genes, GA20ox1 and GA20ox2, in the inoculated sample as well as one GA deactivating gene, GA2ox1. Furthermore, two genes encoding Aux/IAA transcriptional repressors, IAA7 and IAA9, were found up-regulated, while one gene possessing ARF10 was found to be under down-regulation in the inoculated sample. The enhanced activity of these repressor proteins indicates that elevated levels of auxin might be present in biological active form. Furthermore, a number of genes known to show inducible patterns of expression related to auxin hormones were found up-regulated in the inoculated sample, possibly in response to varying regime of these hormones during their interaction. Based on these data, we suggest that the varying levels of both auxin and gibberellins might be crucial to amplify the extent and modulate the levels of ROS in the resistance reaction to nematode invasion. We also believe that the bioactivity of these plant hormones, as well as other defense-related small molecules, might be related to the activity of glycosyltransferases on nucleotide sugars (NDP-sugars). Furthermore, this model reinforces previous ideas that propose the integration of phenolic compounds into the redox system with large dependence on cellular nucleotide sugar concentrations. This latter suggests further effort is needed to investigate pathways that might play an essential role for nucleotide-sugar biosynthesis and for the regulation of the NDP-sugar pool in the host challenged by the RKN– *Meloidogyne* genus in the incompatible interaction.

## Conclusion

We propose that increasing amount of reactive oxygen species (ROS) in the nematode inoculated soybean genotype (PI 595099), when compared to a mock-inoculated control, might has an immediate effect on halting pathogenesis. ROS is thought to activate Ca^2+^ conductance across plant cell membranes probably with the participation of plant annexins found at growth points as root hair. Elevated annexins abundance is hypothesized to positively correlates with auxin-induced ROS accumulation in cells of the quiescent centre reinforcing the view that annexins could function as putative ROS-regulated Ca^2+^ influx pathway [42]. Cell-wall interactions including Ca^2+^ are likely to stabilize pectin networks of interaction causing stimulated pectin synthesis leading to changes in the carbohydrate metabolism. Given the long–term deleterious effects of ROS, the modulation of amplitude and onset of its levels in the host might occur through signaled induction to restore the redox state of the cell compartments. This redox system is thought to have a large dependence on cellular nucleotide sugar and pectic polysaccharides concentrations suggesting that further investigations on pathways that play essential roles for NDP-sugar biosynthesis and for the regulation of the NDP-sugar pool in the host is needed to elucidate its functioning in providing the host with enhanced antioxidant redox buffering capacity. Nucleotide sugar-forming enzymes as sucrose synthase and glycosytransferases are likely important players concerning this aspect. Furthermore we suggest that host coordinate and modulate defenses mostly by the interplay between Auxin (AUX), Gibberellin (GA) and Jasmonate (JA) mediated signaling pathways. Activity of glycosyltransferases such as UDP-dependent glycosyltransferases (UGTs) on the NDP-sugar pool is suggested as important players in the bioactivity of these plant hormones and signaling crosstalk.

GAs are thought to be essential to the observed successful defense outcome. We suggest that a DELLA-like protein induced upon pathogen attack acts integrating signals from auxin. GA and JA in both growth and defense response processes causing ROS levels to remain low. Therefore, the varying levels of GA through mediated signaling are thought strongly to influence the outcome of the plant’s responses to stress and the defense reaction, including establishment of effective systemic immunity in the plant-pathogen interactions.

Although aware that the absence of sequenced mRNA sample replicates (technical or biological) implies in limitations to test differential expression based on sequence data, we presented further indications that the sequence data regarding the “gene counts” fitted very properly. This study provides insights into the incompatible soybean-RKN interaction and suggests that the varying levels of GA through mediated signaling mainly by auxins are thought to strongly influence the outcome of the plant’s responses to stress and the defense reaction, including establishment of effective immunity in the plant-pathogen interactions. A set of genes identified by transcriptional profiling analysis of sequencing data will be tested further for their role in this biological process and can be a useful resource for broadening plant resistance to root-knot nematodes.

## Methods

### Plant material

Resistant and susceptible soybean genotypes to *M. javanica*, PI 595099 (PI) and BRSMG 250 ‘Nobreza’, respectively, were used to assess the RKN-induced changes during incompatible and compatible reactions. The main resistance sources that compose PI 595099 pedigree are shown in Additional files 5 and 6.

### Nematode inoculation

Soybean seed was sown in sterilized sand (120°C for 30 min) and germinated in an acclimatized chamber under a 16-hour-photoperiod at 27 ± 2°C. After a 72 h period the plantlets were transplanted to test pots containing 300 mL of sterile substrate (2 soil: 1 sand). Eight days after transplanting the soybean plants were inoculated with 500 J_2_ of *M. javanica* per plant.

### Histological experiments

Five infected soybean root samples were randomly collected from each time point (1, 2, 4, 6 and 8 DAI) and stained with industrial food colorant [69]. The presence of juveniles in the roots was registered through a stereo microscope (SQF-F; Tecnival, Argentina) with a 30x magnification and statistically analyzed according to a generalized linear model (GLM) with Poisson error as a function of time point and genotype. In addition, gall number, egg masses and total population were estimated after 45 days of inoculation in each genotype in order to evaluate the nematode reproduction. The experiment was performed as a randomized complete block design with five replications per period. The averages between genotypes were compared in relation to gall number, egg masses and number of J_2_ and eggs (total population). Statistical analysis was carried out using the free R programming language (http://www.r-project.org/) with significance level of 5%, and the averages were compared using Kruskal-Wallis non-parametric method.

### Pyrosequencing

Root sections of five independent biological replicates, from soybean resistant line PI 595099 inoculated and mock-inoculated with 500 J_2_ of *M. javanica*, were collected at each time point (0, 6, 12 h, 1, 2, 4, 6 and 8 DAI). Mock-inoculated roots were treated the same as inoculated roots except no J_2_ nematodes were added and RNA samples were taken from soybean roots of both treatments. Tissues of all time intervals were pooled and total RNA was extracted using Trizol reagent (Invitrogen Life Technologies, Ambion®, UK) according to the manufacturer’s instructions. The single RNA pool of both inoculated and mock-inoculated control samples was then subjected to large-scale pyrosequencing using a 454 GLX titanium sequencer employing a single run. We deposited the raw sequence data in SRA, under accession number SRA069880. Transcript sequences cited in the manuscript are already included in the public database Phytozome, *Glycine max* reference genome release 1.0 (http://www.phytozome.net).

### Assembly of transcript reads

Raw 454 sequencing reads were processed with est2assembly pipeline [16] and the assembly of transcript reads was carried out by a guided assembly approach using the soybean reference genome [70]. Mapping against the reference genome was done using the PASA software [17]. Additionally, for the non–aligned EST sequences a *de novo* transcript assembly was performed with Mira Assembler 3.4.0 using default parameters and the resulting clusters were used as input for a second PASA run.

### Gene expression analyses

Gene expression analysis was carried out using the “gene counts” obtained by summing the number of sequences mapping to exons within each gene model in the Glyma 1.0 Soybean reference genome release at Phytozome. For genes with multiple transcripts, we took only one transcript to represent the gene. For the contrast of the gene counts between inoculated and mock-inoculated samples we applied a statistical test implemented in the glm edgeR software [20,71], which uses a negative binomial distribution to model the digital gene expression across conditions based on generalized linear models (glms) suitable for multifactor experiments of any complexity. Genes possibly undergoing transcriptional changes were selected within a raw p–value cutoff of 0.1 and imposing that the sum of the gene counts to each gene were greater or equal to 5.

Aware that the absence of sequenced mRNA sample replicates (technical or biological) implies in limitations to test differential expression based on sequence data we looked for indications that the sequence data regarding the “gene counts” fitted very properly by 1) carry out a descriptive analysis of the sequence data to obtain indications that those gene counts were, for each sample, correlated at some acceptable level so that the comparisons across treatments within the only one sequencing run show small deviations from uniformity; 2) admit a reasonable estimation of global biological variation (BCV) across all genes, then insert these estimation as the dispersion (BCV^2) into the edgeR data object containing the experiment design matrix; 3) proceed to statistical model fitting, and 4) isolate a large subset of genes based on raw p-values to perform a ranked analysis of fold changes in association with gene ontology (GO) terms.

### Functional annotation

Comparative analyses for functional annotation were carried out using the soybean gene coordinates taken from Phytozome v7.0. When necessary, manual annotation of selected genes was performed by transferring best similarity search results using the program BLAST [72] against Arabidopsis proteins obtained from The Arabidopsis Information Resource blast datasets (TAIR 10). For selected gene families specialized databases, like Pfam [73] and PeroxiBase [44], were used to improve annotation.

Mapping between Phytozome’s gene model identifiers and GO terms was performed using *Glycine max* GO annotation file downloaded from agriGO download center [74] and Biomart resource at Phytozome. FUNC package [21] was used for detecting significant associations between PI 595099 gene sets and GO annotations. Additional redundancy removal and visualization of significant associations was performed using the web tool REViGO [22].

In order to capture prominent functional patterns we also categorized the set of differentially expressed genes based on the presence of domains annotated according to Pfam classification [73] using the InterproScan software. Pfam signatures were mapped to GO terms to detect cases of strict functional implications of sets of predicted domains using the Pfam2GO mapping of external classification systems to GO provided by the Gene Ontology Consortium [75]. When the mapping was not obtained thorugh these means, we used data about recorded unintegrated domains as provided by SuperFamily [76] or Panther [77] databases to infer the GO annotation.

### Internet resources

Phytozome, http://www.phytozome.net (May 23, 2011)

Cytochrome P450 database, http://drnelson.uthsc.edu/CytochromeP450.html (May 23, 2011)

PeroxiBase, http://peroxibase.toulouse.inra.fr (May 23, 2011)

PredGPI, http://gpcr2.biocomp.unibo.it/gpipe (May 23, 2011)

SignalP, http://www.cbs.dtu.dk/services/SignalP (May 23, 2011)

agriGO, http://bioinfo.cau.edu.cn/agriGO (May 23, 2011)

REViGO, http://revigo.irb.hr (May 23, 2011)

Soybase, http://www.soybase.org (Oct 31, 2012)

## Competing interests

The authors declare that they have no competing interests.

## Authors’ contributions

MELS prepared nematodes inoculums. MELS, MAC and MJC carried out the time course experiments of RKN. JPD performed the statistical analyses of histological experiments. MAB and RMSA carried out preparation of RNA for 454 sequencing. OBSJ, GJP and RCT analyzed the sequence data and interpreted the data. OBSJ, MAB, MELS and EVSA drafted the manuscript. MAB, EVSA, MCMS and AAPF edited the manuscript. MFGS conceived and coordinated the study. All the authors read and approved the final manuscript.

## Supplementary Material

Additional file 1 **Gall number and egg masses per root milligram.** Number of galls and egg masses at 45 DAI after inoculation of *M. javanica* J_2._. Bars followed by the same letter do not differ significantly at P ≤ 0.05 according to Scott & Knott test. Click here for file

Additional file 2 **Analyses of reproduction factor of *M. javanica*.** Number of juveniles and eggs (total populations) at 45 DAI. Bars followed by the same letter do not differ significantly at P ≤ 0.05 according to Scott & Knott test. Click here for file

Additional file 3 **Sequence assembly and similarity searches (a) Long-ORFs extraction step allows both complete gene models with a start and a stop codon and partial gene models.** (b) Comparison done with Glyma1.0 gene models available at Phytozome. Click here for file

Additional file 4 **A) List of genes along with the differential expression quantification showing correspondence with at least one GO term annotation found in statistical significance by the Wilcoxon rank-test.** B) List of genes along with the expression quantification in terms of logFC showing correspondence with at least one SSR marker found in statistical significance to QTL conditioning PI 595099 resistance against. Click here for file

Additional file 5 **Genealogic tree of soybean line PI 595099.** The ancestors presented in the genealogy of the soybean line PI 595099 are published in Crop Science from 1964 to 1997. Click here for file

Additional file 6 **Reaction of the main soybean genotypes used as source of resistance to *Meloidogyne javanica*, *M. incognita *and *M. arenaria *in PI 595099 pedigree. **^1^ Resistant; ^2^ Moderately resistant; ^3^ Susceptible. Click here for file
