# Supplementary figures and images for: Transcription profile of soybean-root-knot nematode interaction reveals a key role of phythormones in the resistance reaction
Source: BMC Genomics. 2013 May 10;14:322. doi: 10.1186/1471-2164-14-322 (PMC3701510; doi:10.1186/1471-2164-14-322)

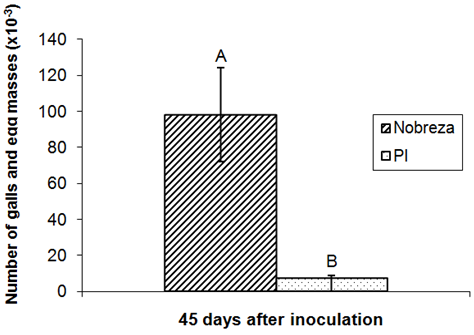

Supplement: Additional file 1 — Gall number and egg masses per root milligram. Number of galls and egg masses at 45 DAI after inoculation of M. javanica J2.. Bars followed by the same letter do not differ significantly at P ≤ 0.05 according to Scott & Knott test. [file 1471-2164-14-322-S1.tiff]

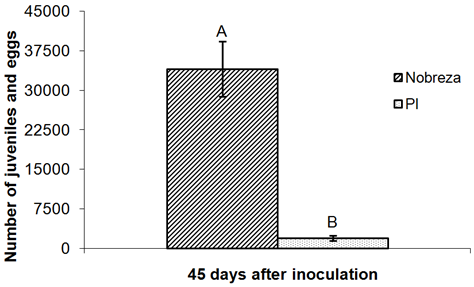

Supplement: Additional file 2 — Analyses of reproduction factor of M. javanica. Number of juveniles and eggs (total populations) at 45 DAI. Bars followed by the same letter do not differ significantly at P ≤ 0.05 according to Scott & Knott test. [file 1471-2164-14-322-S2.tiff]

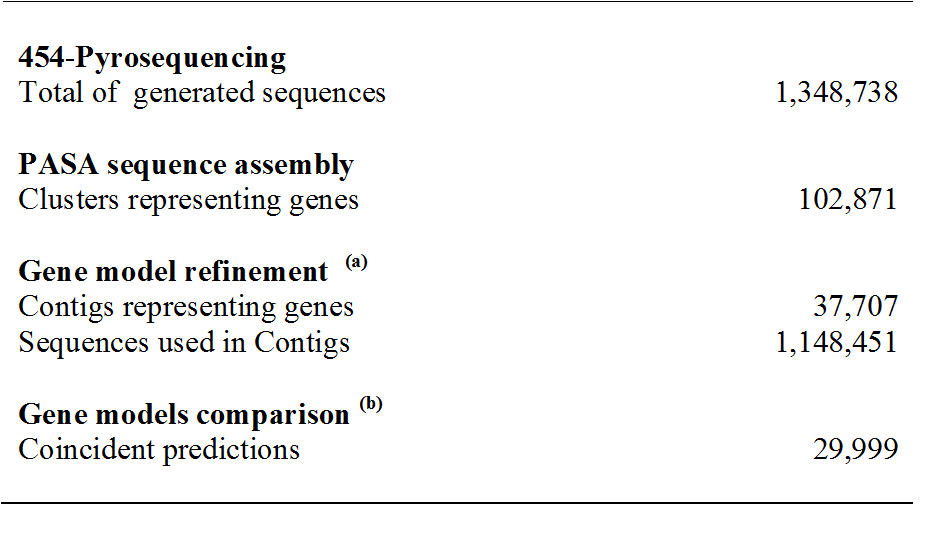

Supplement: Additional file 3 — Sequence assembly and similarity searches (a) Long-ORFs extraction step allows both complete gene models with a start and a stop codon and partial gene models. (b) Comparison done with Glyma1.0 gene models available at Phytozome. [file 1471-2164-14-322-S3.tiff]

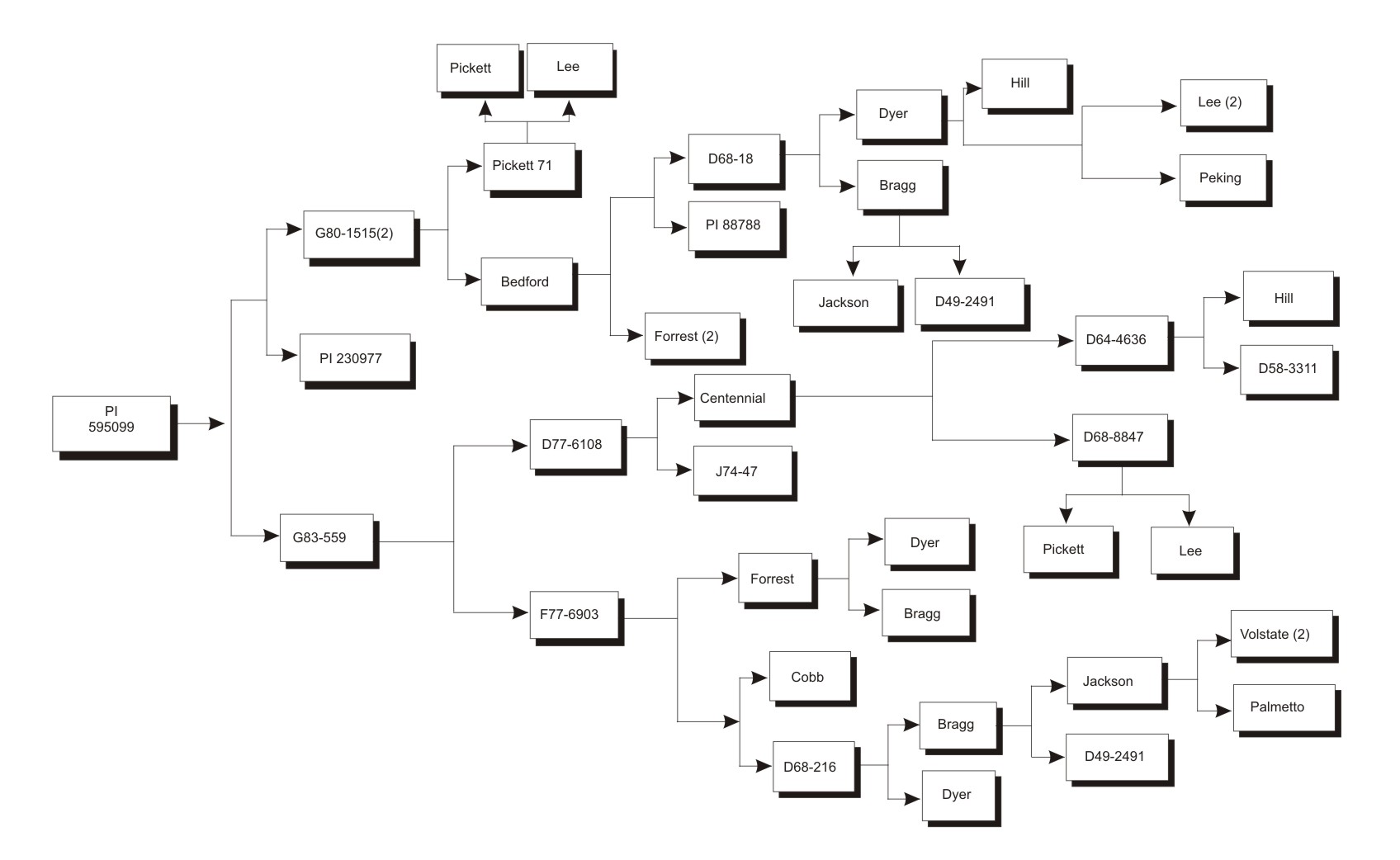

Supplement: Additional file 5 — Genealogic tree of soybean line PI 595099. The ancestors presented in the genealogy of the soybean line PI 595099 are published in Crop Science from 1964 to 1997. [file 1471-2164-14-322-S5.jpeg]

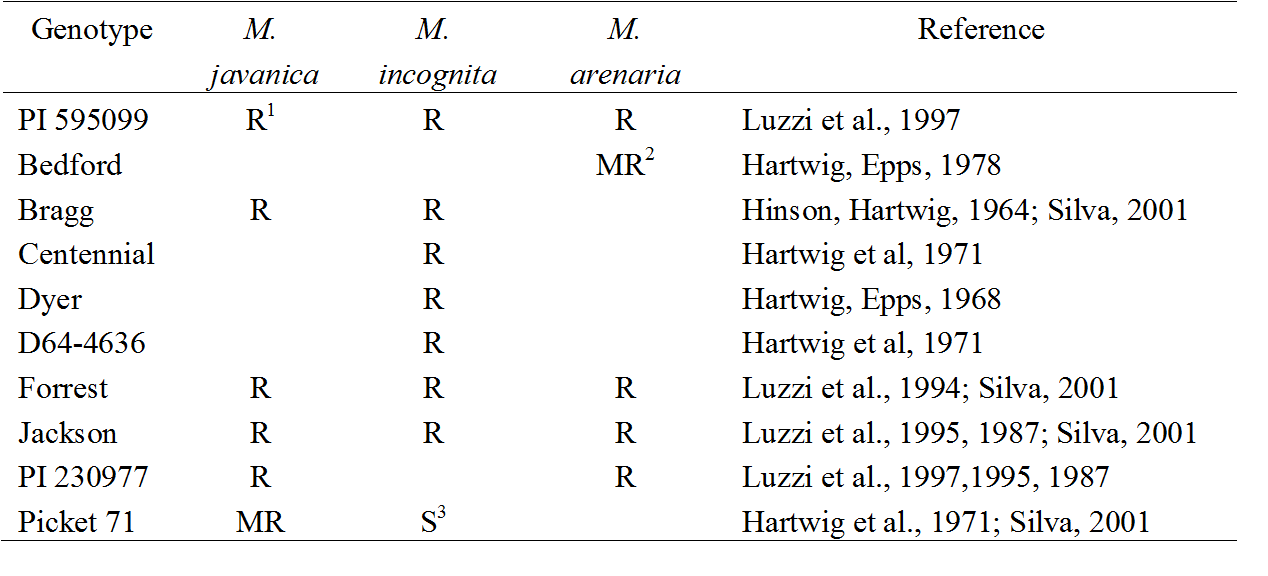

Supplement: Additional file 6 — Reaction of the main soybean genotypes used as source of resistance to Meloidogyne javanica, M. incognita and M. arenaria in PI 595099 pedigree. 1 Resistant; 2 Moderately resistant; 3 Susceptible. [file 1471-2164-14-322-S6.tiff]
